# Evaluation of malaria surveillance system in Kano State, Nigeria, 2013–2016

**DOI:** 10.1186/s40249-020-0629-2

**Published:** 2020-02-10

**Authors:** Tyakaray Ibrahim Visa, Olufemi Ajumobi, Eniola Bamgboye, IkeOluwapo Ajayi, Patrick Nguku

**Affiliations:** 1 Nigeria Field Epidemiology and Laboratory Training Program, Abuja, Nigeria; 2 National Malaria Elimination Program, Federal Ministry of Health, Abuja, Nigeria; 3 School of Community Health Sciences, University of Nevada, Reno, USA; 4 Department of Epidemiology and Medical Statistics, College of Medicine, University of Ibadan, Ibadan, Nigeria; 5 African Field Epidemiology Network Nigeria Country Office, Abuja, Nigeria

**Keywords:** Malaria, Performance, Operation, Surveillance system attribute, Health management information system, Nigeria

## Abstract

**Background:**

Malaria surveillance system strengthening is essential in the progress towards malaria elimination. In Nigeria, more attention is being given to this recently as the country is striving towards achieving elimination. However, the surveillance system performance is fraught with challenges including poor data quality with varying magnitude by state. This study evaluated the operation of the Kano State malaria surveillance system and assessed its key attributes.

**Methods:**

An observational study design comprising a survey, record review and secondary data analysis, and mixed methods data collection approach were used. Four key stakeholders’ and 35 Roll Back Malaria Focal Persons (RBMs) semi-structured interviews on operation of the system and attributes of the surveillance system, were conducted. We analyzed the abstracted 2013–2016 National Health Management Information System web-based malaria datasets. The surveillance system was evaluated using the “2001 United States Centers for Disease Control’s updated guidelines for Evaluating Public Health Surveillance Systems”. Data were described using means, standard deviation, frequencies and proportions. Chi-squared for linear trends was used.

**Results:**

Overall, 24 RBMs (68.6%) had ≤ 15-year experience on malaria surveillance, 29 (82.9%) had formal training on malaria surveillance; 32 RBMs (91.4%) reported case definitions were easy-to-use, reporting forms were easy-to-fill and data flow channels were clearly defined. Twenty-seven respondents (69.2%) reported data tools could accommodate changes and all RBMs understood malaria case definitions. All respondents (4 stakeholders and 34 RBMs [97.1%]) expressed willingness to continue using the system and 33 (84.6%) reported analyzed data were used for decision-making. Public health facilities constituted the main data source. Overall, 65.0% of funding were from partner agencies. Trend of malaria cases showed significant decline (*χ*^*2*^_*trend*_ = 7.49; *P* = 0.0006**)**. Timeliness of reporting was below the target (≥ 80%), except being 82% in 2012.

**Conclusions:**

Malaria surveillance system in Kano State was simple, flexible, acceptable, useful and donor-driven but the data were not representative of all health facilities. Timeliness of reporting was suboptimal. We recommended reporting from private health facilities, strengthening human resource capacity for supportive supervision and ensuring adequate government funding to enhance the system’s representativeness and improve data quality.

## Background

In 2018, malaria remains a public health threat with estimated 228 million cases and 405 000 malaria deaths reported globally and Africa accounting for 93% of the cases and 94% of deaths [1]. In Nigeria, about 57.2 million malaria cases and 95 844 malaria deaths were recorded [2]. Malaria transmission is meso-endemic in Kano State, Nigeria with a prevalence of 32% [3]. Nigeria is currently in the malaria control phase, but efforts are being intensified to transit to elimination phase. Effective and responsive malaria surveillance system is essential in the control and elimination of malaria.

Public health surveillance is the ongoing, systematic collection, analysis, interpretation, and dissemination of data regarding a health-related event for use in public health action to reduce morbidity and mortality and to improve health [4]. Evaluating the malaria surveillance system is important to ensure quality data are used for generating information needed for planning, targeting interventions and monitoring malaria programs [5].

The ability of a surveillance system to detect outbreaks and monitor epidemiologic trends depends on its sensitivity, which is the proportion of all diagnosed cases of a disease that is identified by the surveillance system. A system with high sensitivity identifies challenges and gaps which would guide allocation and targeted deployment of appropriate resources for disease control. Improvement in a country’s surveillance system will aid ascertaining trend in the burden of the disease and thus need for periodic performance evaluation of the system [6]. Perfect surveillance system is desirable though it might not be attainable in an effort to eliminate malaria. However, persistent gaps in the system precludes optimal deployment of resources for malaria elimination. Additionally, improvement in country’s surveillance system will aid tracking of progress towards elimination. Essentially, a higher standard of surveillance system is desirable for malaria elimination.

According to the World Health Organization (WHO) malaria surveillance and monitoring reference manual, all major components of a malaria surveillance system should be integrated into broader health management information systems (HMIS), including, where applicable, systems for reporting notifiable diseases [5]. In 2013, the pre-existing malaria specific information system was integrated into the National Health Management Information System (NHMIS) in Nigeria. In some settings, a vertical system may be used initially, but it should allow communication with and an eventual integration into the HMIS for sustainability. The HMIS system should in turn, be responsive to the promptness of data required for effective malaria surveillance [5]. Therefore, quality information systems are necessary as they are important instruments for malaria control and elimination [6]. Data from information systems identify populations under risk thereby suggesting specific population to target for intervention [7].

Evaluation of a surveillance system is the systematic investigation of the merit, worth or public health significance of the surveillance system. The purpose of evaluating public health surveillance systems is to ensure that problems of public health importance are being monitored efficiently and effectively [4]. Additionally, evaluation helps to determine if a system is meeting the set objectives and whether the attributes are efficient to achieve these objectives. The evaluation of public health surveillance systems should involve an assessment of system attributes, including simplicity, flexibility, data quality, acceptability, sensitivity, predictive value positive, representativeness, timeliness, and stability [4].

Nigeria operates a federal system of government under three arms, namely the Executive, the Legislative, and the Judiciary. It is made up of 36 states and a Federal Capital Territory. The country has both government-owned (public) and private-owned health facilities. There are three tiers of public health facilities (primary, secondary and tertiary). Kano State was selected for this evaluation due to its high prevalence of malaria (currently 32%) [3] and the recent unusual increase in number of malaria cases reported some months prior to this evaluation [8]. In 1998, the malaria surveillance system in Kano State was established and hitherto, it had been detecting cases. However, some of the attributes of the system are not known. The objectives of the system are to detect changes in trends of malaria cases, estimate morbidity and mortality due to malaria infection and assess impact of control measures. We conducted an evaluation to describe the operations of the Kano State malaria surveillance system, assess its key attributes and performance of the system in line with its set objectives.

## Methods

### Study setting

Kano State lies between latitude 13° N and 11° S and longitude 8° W and 10° E. It occupies land area of 20 760 km^2^ and is located in North-Western Nigeria, West Africa. The state has 44 local government areas (LGAs). The temperature of Kano ranges between 15.8 °C and 33 °C although sometimes during the harmattan it falls to as low as 10 °C. Kano has two seasonal periods; 5 months of wet season (May to September) and 7 months of dry season (October to April). The average rainfall ranges from 63.3 mm ± 48.2 mm in May to 133.4 mm ± 59 mm in August. Malaria is endemic and its transmission peaks between September and February, thus reflecting the climate and suitability to support mosquito breeding and malaria parasite transmission [9]. Overall, 1186 health facilities exist in the state comprising 1045 primary health care facilities, 33 secondary health care facilities, 2 tertiary health care facilities and 106 registered private health facilities. Malaria treatment is free for children below 5 years and pregnant women. The treatment guideline indicates artemisinin-based combination therapy (ACT) as the first drug of choice for treatment of uncomplicated malaria. Preventive measures such as long-lasting insecticidal nets and indoor residual spray are deployed in the state [10]. The State Malaria Elimination Program is responsible for malaria control activities. Similarly, LGA Roll Back Malaria focal persons are responsible for their respective LGAs.

### Study design

An observational study design was used, and this comprised a survey, record review and secondary data analysis. The survey was conducted among Roll Back Malaria focal persons (RBMs) in Kano State, Nigeria. Retrospective review and analysis of 2013–2016 web-based NHMIS malaria data was conducted.

### Data collection

This evaluation was conducted in December 2016. Mixed methods data collection approach was used (Additional file 1). A standardized semi-structured questionnaire was administered to thirty-five purposively selected RBMs from thirty-five LGAs. Information on socio-demographic characteristics, years of experience and attributes of the surveillance system was collected. The RBMs were selected based on their experience and role in providing technical assistance to malaria surveillance at the LGA level.

Key informant interviews (KII) were conducted with four stakeholders (state malaria program manager, state monitoring and evaluation officer, state epidemiologist and the state disease surveillance and notification officer) to obtain their inputs in describing the system and assessing key attributes of the system (Additional file 2). These individuals were targeted for interview to ensure that the eventual findings from the evaluation would be implemented. The questionnaires and key informant interview guide were adapted from the United States Centers for Disease Control and Prevention (CDC), 2001 updated guidelines for evaluation of public health surveillance systems [4]. Malaria surveillance data comprising epidemiological and laboratory variables were abstracted from the January 2013 to December 2016 from the NHMIS, and analyzed to assess and demonstrate some of the attributes of the system such as the timeliness of reporting and reporting rate.

### Data analysis

Findings from both the quantitative and qualitative assessments were described in comparison with standards in the CDC guidelines. Quantitative data from survey and the abstracted NHMIS data were analyzed descriptively using the statistical software Excel 16.0 (Microsoft Corporation, One Microsoft Way Redmond, USA) and Epi-info 7.0 (CDC, Atlanta, USA). Data were summarized using means, standard deviation, frequencies, proportions and presented in tables and charts. Chi-squared analysis for linear trends in proportion was performed for the reported annual malaria cases. Timeliness of reporting was calculated as “number of monthly reports received from health facilities within stipulated time period (on or before the 5^th^ day of the new month) as a proportion of expected total number of health facility reports”; this was calculated for each year. Reporting rate was calculated for each year as “number of monthly reports received from health facilities as a proportion of expected total number of health facility reports”. Qualitative assessment was performed by measuring key indicators such as changes in the system (whether adapted or not), data reporting (data entry, accessibility and validation), training and supportive supervision (whether integrated or malaria focused) and funding sources (government or partner driven). The KII were analyzed thematically based on questions from the guide and similar responses were clustered.

## Results

### Demographic characteristics

**Fig. 1:**
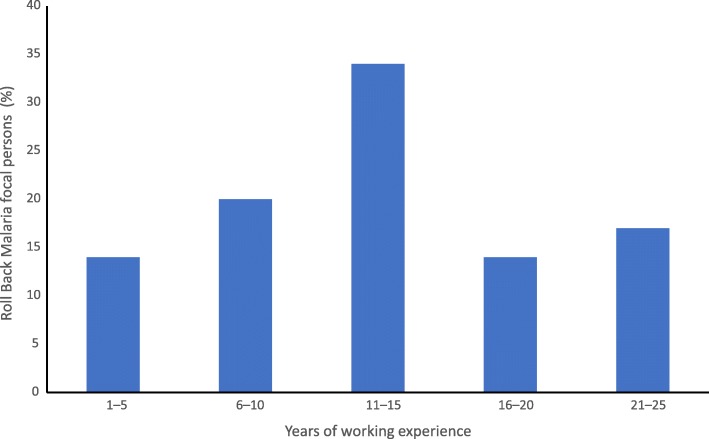
Years of work experience in malaria surveillance by Roll Back Malaria focal persons in Kano State

Overall, 35 RBMs were interviewed. Mean age of the RBMs was 28 ± 2 years. Twenty (57.1%) were less than 30 years. Twenty-four (68.6%) had ≤ 15 years of work experience in malaria surveillance unit (Fig. 1). Thirty-two (91.4%) of the RBMs were community health extension workers and the rest were environmental health officers; 29 (82.9%) were formally trained on malaria surveillance activities.

Data from the 2013 to 2016 NHMIS had 2 164 759 fever cases, of which 1 285 271 (59.4%) were confirmed malaria cases. Of these confirmed cases 552 755 (43.0%) were < 5 years and 732 516 (57%) were ≥ 5 years. Of the 732 516 cases, 96 584 (13.2%) were pregnant women.

### Description and operation of the malaria surveillance system in Kano state

The surveillance system in Kano State follows that of the other states in the federation and consists of stakeholders at the state and LGA levels involved with information collection, collation and planning for the effective operation of the system. The actors include the malaria program managers at state and LGA levels who offer technical assistance and the monitoring and evaluation (M&E) officers who do the actual data entry into district health information system version 2 (DHIS2), the web-based platform for NHMIS. Data flow from the health facility level where service providers record data of services provided into NHMIS registers and collate these into NHMIS-monthly summary forms; The LGA M&E officers collate these data from all health facilities and enter the data into the DHIS2 platform. The data can only be accessed by designated stakeholders at various levels where data are used for decision making and feedback is given to the various levels in the system.

**Fig. 2:**
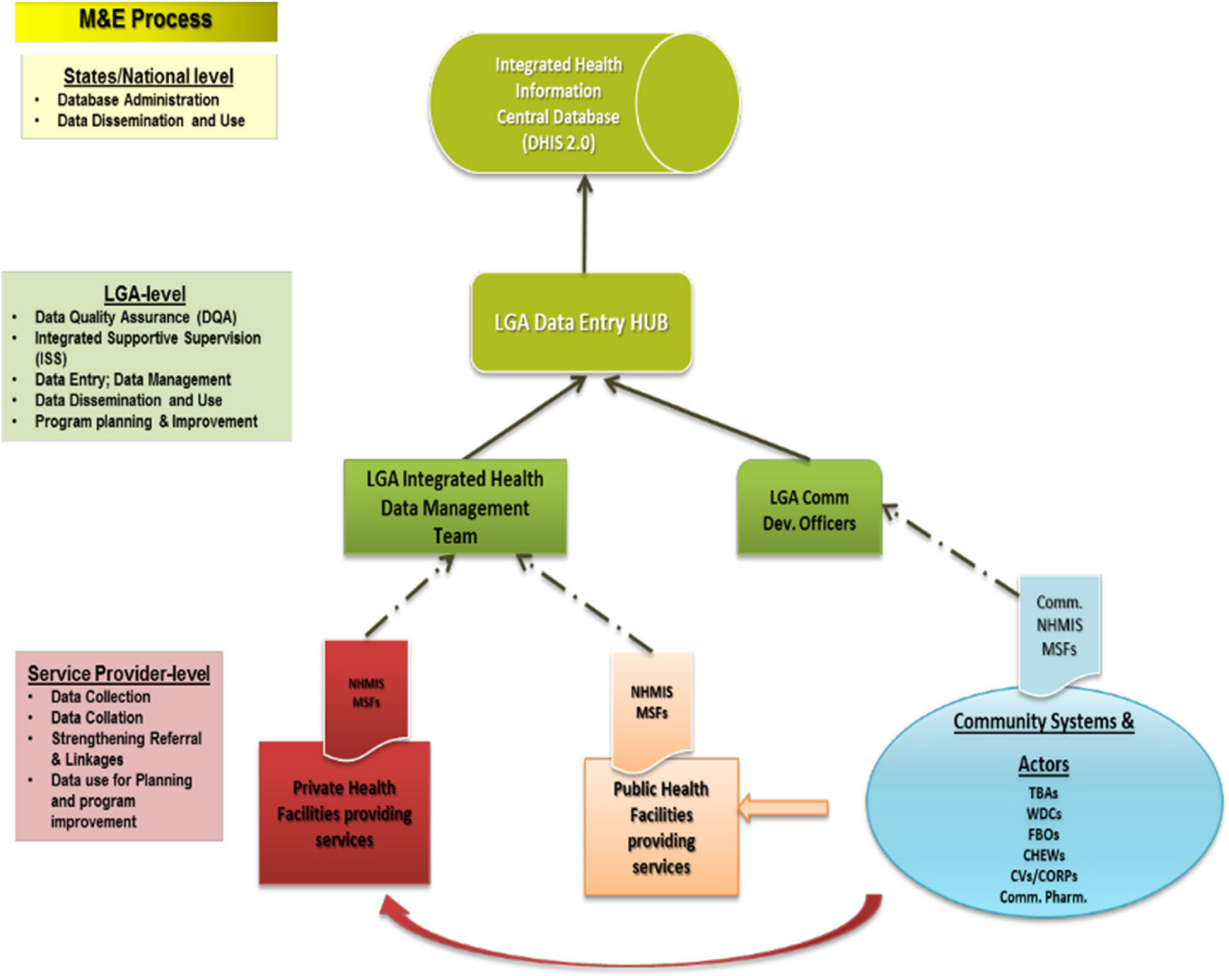
Operation of the malaria surveillance system in Kano State, Nigeria. M&E: Monitoring and evaluation; NHMIS: National Health Management Information System; MSFs: Monthly summary forms; TBAs: Traditional birth attendants; WDCs: Ward Development Committees; FBOs: Faith-Based Organizations; CHEWs: Community health extension workers; CV: Community volunteers; CORPs: Community oriented resource persons; Comm. Pharm.: Community pharmacist; LGA: Local government area; HUB: Common data connection point; Dev.: Development; Comm: Community

Malaria surveillance system in the context of NHMIS showed passive malaria surveillance, the routine data collection on malaria cases at health facility level all year round (Fig. 2).

### Kano state malaria surveillance systems attributes

#### Usefulness

**Fig. 3:**
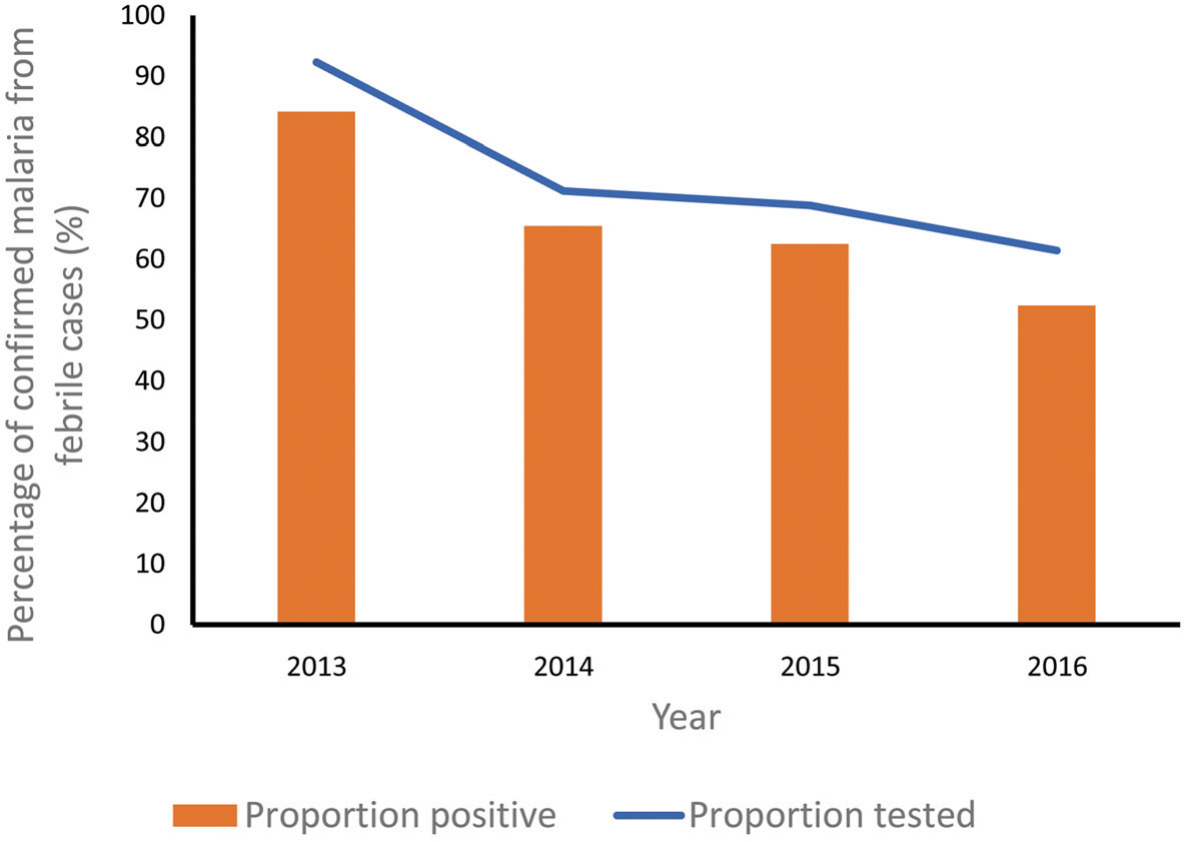
Trend of confirmed malaria and testing rates of fever cases in Kano, Nigeria 2013–2016

**Fig. 4:**
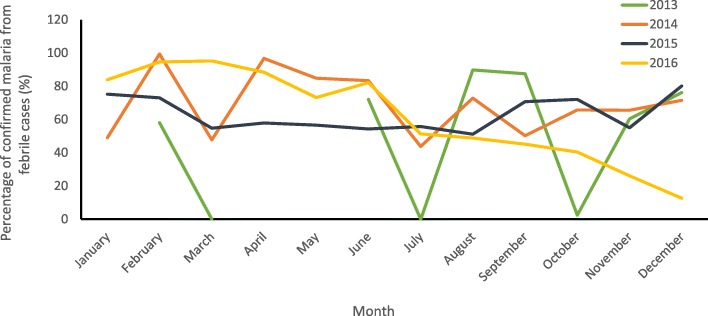
Monthly trend of confirmed malaria cases in Kano State, January 2013*–*December 2016

The malaria surveillance system was set up to detect malaria epidemics promptly, especially in areas with seasonal increase in clustering of malaria cases or with a large population at risk. The trend in malaria cases seen over the period of data review was described (Fig. 3) The system has been useful in detecting cases over the years. There was a sharp decline from 83% in 2013 to 62% in 2014, thereafter the decline plateaued between 2014 and 2015 followed by further decline from 60% in 2015 to 48% in 2016. The decline in cases was statistically significant (*χ*^***2***^_***trend***_ **=** 7.49; *P =* 0.0006**)** and consistent with testing rates by Rapid Diagnostic Kit (RDT) and microscopy (Fig. 3). The RBM focal persons reported interruptions of the supply of malaria commodities in the state. Monthly report of confirmed malaria cases was missing for 5 months in the year 2013, which was probably because the DHIS was newly introduced in the second quarter of that year. Irregular pattern of monthly malaria trend was observed across the years of review (Fig. 4). In all, 33 (84.6%) respondents reported analyzed data were used for decision-making.

#### Simplicity

Thirty-two RBMs (91%) described the system as simple, mentioned the case definitions were well understood at all levels, standardized electronic and paper-based tools were in use, reporting forms were easy-to-fill, communication channels between all levels were well established and data flow was clearly defined. Data was collected using the LGA summary form and 30 RBMs (85.7%) indicated the tool was easy to fill, this was corroborated by all the state level stakeholders. All the respondents indicated that uploading data into the DHIS2 was not difficult.

#### Flexibility

Overall, 24 (61.5%) RBMs and stakeholders stated that allowance for changes in data tools and reporting sources have been accommodated in the data collection tool with minimal influence on workload. Twenty-five (64.1%) RBMs and stakeholders indicated that modifications in the system were included within the first months of 2013 and 27 (69.2%) mentioned they have been able to adjust to the changes made. The system adapted well to the newly revised national standard operating procedure for malaria surveillance, monitoring and evaluation as it accommodated new variables and information such as the daily outpatient department case register for management of malaria which was modified to capture date of onset of fever. Similarly, the changes in the national diagnosis and treatment guidelines which was formally based on presumptive clinical diagnosis and treatment with monotherapy is now based on parasitological diagnosis and treatment with ACT. This was affirmed by key stakeholders, who attested to the smooth transition from the old data tool to the new one and the major resource used to achieve this was human resource.

#### Acceptability

All the respondents were willing to continue with the data collection process; case definitions and tools were acceptable to all stakeholders. Thirty-three (33/39, 84.6%) have been fully involved in the malaria surveillance system and expressed that the system appreciates their efforts in carrying out their job.

#### Representativeness

Data for the period evaluated were collated essentially from primary and secondary public health facilities; data from the two tertiary hospitals and all private health facilities were excluded, thus the surveillance system was not representative of all health facilities in Kano State. All RBMs attested to the fact that the data tools used in the system captured information on distribution of cases of malaria based on age, sex, location, outcome of disease and date of diagnosis.

#### Timeliness of reporting

**Fig. 5:**
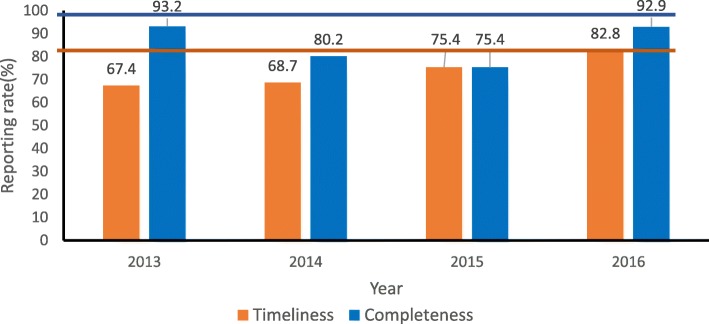
Timeliness and reporting rates of malaria surveillance reports in Kano State, Nigeria 2013*–*2016

In 2016, timely reporting was observed with the annual reporting rate being 82.8% which is higher than the 80% target for timely reporting. However, the timeliness of reporting for earlier years 2013 (67.4%), 2014 (68.7%) and 2015 (75.4%) were below the target. **Reporting rate:** None of the reported data of all the years reviewed met the 100% target for reporting rate (Fig. 5).

#### Stability

There has not been replacement of RBM focal persons (the LGA level personnel) with new individuals during the evaluation period. Data collected were being managed appropriately with fully functional computers; 68% of the RBMs indicated stock-out of malaria commodity as one of the major challenges faced. Key stakeholders indicated 65.0% of the funding was from partner agencies, 32.4% from state government and 2.6% from local government.

From the KII, stakeholders mentioned that there was irregular supply of RDT kits, other data tools and guidelines for malaria management. On data quality, the state had conducted series of data improvement training at least once a year. Integrated supportive supervision to health facilities was conducted weekly, and this was said to have helped to improve quality of malaria data in the state. Stakeholders also indicated the need for frequent capacity building and more staff to assist with data management.

## Discussion

Malaria surveillance system in Kano State was found to meet some of the attributes of a good surveillance system. The state-level stakeholders (state malaria program officer, state epidemiologist, state malaria M&E officer and the state disease surveillance and notification officer) and LGA-level health workers (RBMs) attested to the ease of operation of the system which is well structured and flexible to changes. The ease of operation of the system could be as a result of the simplified channels of communication and data tools which was affirmed by the RBM focal persons. This finding is similar to that of studies conducted in Oyo State, Nigeria and in Chipinge District, Zimbabwe where the surveillance systems were adjudged to be simple and flexible [11, 12]. However, it is in contrast to studies in Brazil, Zimbabwe and Angola where the systems were complex to operate [13, 14]. The Kano State surveillance system harnesses a lot of resources partner organization such as Global Fund (through Achieving Health Nigeria Initiative) and United State Agency for International Development and some from the state government, and this aided the smooth operation of the system. Previous study in Oyo State, Nigeria showed majority of funding was also from partner agencies [11].

Malaria commodities are supplied periodically by the federal government and partner organizations to the state for onward distribution to health facilities. Although the surveillance system does not capture commodity supply, the intermittent decline may be attributed to interruptions of the supply of malaria commodities in the state, which was alluded to by the RBM focal persons during the survey. This is akin to observation of a study in Kaduna State where there was an irregular trend of malaria cases due to interrupted supply of malaria commodities [15]. Thus, this emphasizes the importance of consistent supply of malaria commodities and the need to educate clients and health workers on adequate and appropriate utilization of such commodities. There was a decline in malaria cases from 2013 to 2016, which showed the usefulness of the system in detecting malaria cases as shown in a study in Kaduna State [16].

The findings of this study showed that data collection tools were flexible to modification and could accommodate changes in the malaria data capturing. These tools have been fully adopted into them surveillance system and adequately utilized. This is similar to findings from Chipinge district of Zimbabwe and in Ebonyi State, Nigeria where the systems were able to accommodate and adapt to changes [12, 17]. On a closer observation of the older version of NHMIS outpatient department health facility register there were no separate columns for malaria diagnosed using RDT and those diagnosed using microscopy. Both malaria RDT and microscopy results are considered diagnostic of malaria case. The data collection tool has been expanded to include separate columns for diagnosis by RDT, microscopy and overall diagnosis using both methods attesting to the flexibility of the system. These changes have influenced the surveillance of malaria at health facility level as accurate number of confirmed cases are currently available which is essential for ascertaining the true malaria burden.

The malaria surveillance system in Kano State collates data that are not representative of all health facilities as data from the two tertiary health facilities and all private facilities were not included in the general state data. This could be because stakeholders from these facilities are not part of the monthly review meetings of all state level stakeholders and majority of these facilities do not have the updated data tools for reporting. Previous studies in Kaduna State, Nigeria also showed that the malaria surveillance system excluded data from private health facilities [16]. This implies that data from private facilities are not harmonized into the routine reports for the state which can affect inferences and programmatic planning. This is more so as 42.5% [2] of malaria cases are treated in the private sector. Although the proportion of symptomatic cases that were found to seek care and corresponding testing rates reduced over the years, the lack of integration of the private sector within the surveillance system invalidates the accuracy of the data reported for the state, as the data were solely from the public health facilities-based surveillance system, and thus the possibility of underestimation. In a global landscaping of system performance 2015–2017, the common gaps identified across countries include inadequate coverage of the system especially in remote communities and the private sector [6]. In addition, the landscaping found poor integration of data from other sources such as intervention information, poor visualization of generated information, and its lack of availability for making programmatic decisions to be an important gap in the surveillance system globally. In Kano State, our study findings showed that data from the surveillance were used for decision making and formulation of policies that guides the system’s operation.

Furthermore, currently in Kano State there is a structure in place to ensure quality assurance of data captured in the system. The state has a functional state data control room which review all NHMIS data of preceding month within the last 2 weeks of succeeding month, shares this with zonal M&E officers (the LGAs are distributed into six zones) who then addresses this with LGA M&E officers. Additionally, the LGA M&E officers request for clarification of inconsistencies from officers-in-charge/head of each affected health facility and make necessary corrections in the DHIS2 afterwards. Data quality assessment is planned to be conducted every quarter of the year. However, though conducted periodically, it was irregular, and development partner driven (mainly support was from Global Fund and United Kingdom Department for International Development – funded support to National Malaria Program). None of the reported data of all the years reviewed met the 100% target for reporting rate. This has been reported in studies conducted in Kaduna State, Nigeria and Brazil, where the target for reporting rates of data were not attained [16, 18]. The target for timeliness of data reporting was achieved only in the year 2016 across all the years of evaluation. This is similar to findings from Oyo State, Nigeria, where the target for the timeliness of reporting was not attained [11]. However, a study in Ebonyi State, Nigeria in 2014 and in Iran showed a high rate of timely reporting due to set deadlines that were followed. Timeliness of reporting is essential for appropriate programmatic planning and decision making [5, 17]. Several reasons have been adduced to explain the untimely reporting onto the DHIS2 system in Nigeria including delayed submission from health facilities, cumbersome nature of registers, attrition of trained staff, inadequate human resource and erratic internet bandwidth.

The conduct of integrated supportive supervision and on-the-job training (on-site technical assistance whereby health workers are supervised and trained while doing their job on quarterly basis by Kano State Malaria Program staff and all partners) impacted on the improved data quality seen in this study from the monthly trend of reported cases. The finding is similar to that of a study in Kaduna State where good supportive supervision was observed [16], and this emphasizes the importance of adequate supervision as an integral part of an effective surveillance system.

The study has a few limitations. The sensitivity and predictive positive value attributes could not be ascertained. The surveillance data collection tool does not allow for serial testing of malaria cases with both RDT and microscopy as each of these is considered diagnostic of a malaria case and not a screening tool as per the Nigerian national malaria diagnosis and treatment guidelines. Also, the chi squared for trend analysis is highly sensitive to size, which makes association in monthly trend difficult to ascertain due to small proportion of confirmed cases.

## Conclusions

Surveillance is an important tool in the progression from control to elimination of malaria which is the current goal of the Federal Republic of Nigeria. The findings of this evaluation in Kano State revealed the surveillance system was found to be useful, simple, flexible and acceptable but there is a need for improvement in the system’s representativeness, data reporting rates and timeliness of reporting. Representativeness is vital for planning and implementing targeted interventions and measuring progress towards elimination. Having representative figures on coverage of cases tested and treated for malaria, will enable adequate quantification and equitable distribution of commodities. The findings from this study have been shared with key stakeholders in the state malaria surveillance system in order to address all the identified gaps. Moreover, we recommend that the Kano State Ministry of Health should ensure tertiary and private health facilities are included in the malaria surveillance system to enhance its representativeness and stakeholders should provide frequent supportive supervision to improve reporting rates and timeliness of reporting.

## Supplementary information

**Additional file 1.** Questionnaire for Roll Back Malaria Focal Persons on malaria Surveillance System Evaluation.

**Additional file 2.** Key Informant Interview Guide on Malaria Surveillance System Evaluation.

## Data Availability

All data generated or analyzed during this study are included in this published article and if any additional data set is needed, it can be made available by author to the publisher at any time.

## Abbreviations

ACT: Artemisinin-based combination therapy
AFENET: African Field Epidemiology Network
CDC: United States Centers for Disease Control
HMIS: Health Management Information System
KII: Key informant interview
LGA: Local government area
M&E: Monitoring and evaluation
NHMIS: National Health Management Information System
RBMs: Roll Back Malaria focal persons
RDT: Rapid diagnostic test
WHO: World Health Organization

## References

[1] World malaria report (2018). World Health Organization (WHO), Geneva.

[2] Nigeria Demographic Health Survey (DHS) (2018). National Population Commission, Nigeria.

[3] Nigeria Malaria Indicator Survey. National Malaria Elimination Programme (NMEP), National Population Commission (NPopC), National Bureau of statistics (NBS), and ICF international. Abuja; 2016. https://dhsprogram.com/pubs/pdf/MIS20/MIS20.pdf. Accessed 4 Jun 2018

[4] Updated guidelines for evaluating public health surveillance systems: recommendations from the guidelines working group. Centers for Disease Control and Prevention, Atlanta, USA. 2001. Accessed 28 July 2019.

[5] Malaria surveillance, monitoring & evaluation reference manual. WHO Global Malaria Programme, Geneva 2018. https://www.cdc.gov/mmwr/preview/mmwrhtml/rr5013a1. Accessed 27 Oct 2019.

[6] Lourenço C, Andrew JT, Peter MA, Justin MC, Deepa P, Darlene B (2019). Strengthening surveillance systems for malaria elimination: a global landscaping of system performance, 2015–2017. Malar J.

[7] Lima C, Schramm J, Coeli CM, Silva ME (2009). Review of data quality dimensions and applied methods in the evaluation of health information systems. Cad Saude Publica.

[8] Ahmad M (2016). Kano blames outbreak of malaria on residents’ refusal to use mosquito nets. Premium Times.

[9] Tukur A (2010). Temporal variation of malaria occurrence in Kano municipal local government area. Bayero J Pure Appl Sci.

[10] Kano State Malaria Elimination Programme, “Kano State Malaria Quarterly Bulletin,” 2017. http://smoh.org.ng/wp-content/uploads/2018/10/Q2-2018_Kano-State-Malaria-bulletin_Final-Version.pdf. Accessed 23 Oct 2019.

[11] Olugbade OT, Ladipo TO, Israel O, Adedire EO, Adedokun B, Ajumobi O (2014). Malaria surveillance system evaluation, Oyo state, Nigeria 2012. Int J Infect Dis.

[12] Kureya T, Chadambuka E, Mhlanga M, Ndaimani A, Makoni P (2017). An evaluation of the malaria surveillance system of Chipinge District. Int J Heal Sci Res.

[13] Maphosa M, Tsitsi PJ, Masuka N, Mungati M, Gombe N, Nsubuga P (2019). Evaluation of the maternal death surveillance and response system in Hwange District, Zimbabwe, 2017. BMC Pregnancy Childbirth.

[14] Dievie DN, Dongala P, Esteves JP, Ventura BF, Muquila A, Ajumobi OO (2017). Evaluation of the meningitis surveillance system in Luanda Province. J Interv Epidemiol Public Healh.

[15] Ibrahim BS, Abubakar AA, Bajoga UA, Nguku PM (2017). Evaluation of the malaria surveillance system in Kaduna state, Nigeria 2016. Int Soc Dis Surveill.

[16] Bajoga UA, Balarabe HS, Olufemi AA, Dalhat MM, Sule IB, Ibrahim MS (2019). Trend of malaria cases in Kaduna state using routine surveillance data, 2011-2015. Pan Afr Med J.

[17] Agboeze J, Nguku P, Nwankwo L, Okeke L, Ajumobi O (2017). Evaluation of malaria surveillance system in Ebonyi. Ann Med Health Sci Res.

[18] Braz RM, Tauli PL, Santelli AC, Fontes CJ (2016). Evaluation of the completeness and timeliness of malaria reporting in the Brazilian Amazon, 2003-2012. Epidemiol Serv Saude.

