# Supplementary material for: Evaluation of malaria surveillance system in Kano State, Nigeria, 2013–2016
Source: Infect Dis Poverty. 2020 Feb 10;9:15. doi: 10.1186/s40249-020-0629-2 (PMC7008566; doi:10.1186/s40249-020-0629-2)
Supplement: Supplementary file 1 — Additional file 1. Questionnaire for Roll Back Malaria Focal Persons on malaria Surveillance System Evaluation. [file 40249_2020_629_MOESM1_ESM.docx]

**EVALUATION OF MALARIA SURVEILLANCE SYSTEM IN KANO STATE**

QUESTIONNAIRE

Greetings sir/ma

My name is Visa Ibrahim Tyakaray from the Nigerian Field Epidemiology and Laboratory Training Program (NFELTP). This questionnaire is to assess the knowledge, attitude and practice of stakeholders in the Malaria Surveillance system. The result of this investigation will assist the Government in strengthening malaria surveillance in the State. All your responses will be treated with utmost confidentiality and participation is voluntary. Please be honest as much as possible in your answers. Thank you.

Date ......./........./2016

**Socio-demographic data**

1. Name (optional).................................................................................................................

2. Phone Number......................................................................................................................

3. E-mail address ....................................................................................................................

1. Sex: a. Male b. Female
2. Age (yrs.): a. 20-29 b. 30-39 c. 40-49 d. 50-59 e. 60 and above
3. Marital Status: Married ( ) Single ( ) Divorced ( ) Widowed ( )
4. Cadre of staff a. Doctor. b. Nurse. c. Environmental Officer d. CHEW
5. Working experience in health facility (yrs.): a.1-5 b. 6-10 c.11-15 d.16-20 e. 21-25 f. 26-30 g.30-35

**Training**

1. Have you ever been trained on any malaria program? Yes No
2. If yes, what type of training (tick one): a. Malaria management b. Data quality improvement c .M /E on malaria d. Others(specify)

**Malaria Diagnosis and Treatment**

1. Are there rapid kits for malaria diagnosis in your LGAs? Yes No
2. Are there guidelines for Management of uncomplicated malaria? Yes No
3. Are there laboratories in the facility where blood microscopy and blood count can be done? Yes No
4. Are the following instruments available at health facilities?
5. Microscope: Yes No
6. Weighing scale: Yes No
7. Thermometer Yes No

**Simplicity**

1. Which kind of form do you use in data collection? ***Please tick all that applies***
2. Patient card
3. Daily health record (Form 1)
4. Health facility summary form (Form 2)
5. LGA summary form (Form 3)
6. State summary form (Form 4)
7. NHMIS
8. Retrieval form
9. what you think about the forms: are they easy to fill? Yes No
10. Is malaria case easy to identify based on clinical symptoms (i.e. the case is easily ascertained): Yes No
11. Are there any other organizations apart from State Malarial Elimination Programme and National Malarial Elimination Programs involved in receiving your reports? Yes No Not sure
12. Do they have special forms you have to fill? Yes No Not sure
13. How many of your staff are involved in malaria data collection? a.1 b.2 c.3 d. 4 e.5 f. > 5
14. How many of the staff do you think will be optimal in the task? a.1 b.2 c.3 d. 4 e.5 f. > 5

**Flexibility**

1. Do you think the forms have been modified in the past to reflect the present data collected? Yes No
2. How long did it take the system to include these modifications in the present data collected (months): a. 1-4 b. 5-10 c.11- 16 d.17-24 e. >24 months
3. What were the resources used to implement these changes? (tick as applied): a. Human b. Financial c. Both d. None

**Data Quality**

1. The clarity of the data collected: Rate from 1 – 5 (1 = poor, 2 = fair, 3 = good, 4 = very good, 5 = excellent) …………………………….
2. Have you been supervised before? Yes No
3. How many times were you supervised within the last six months? a. 1 b. 2 c. 3 d. 4 e. 5 f. >5times
4. Assess the care taken in completing the surveillance forms: Rate from 1-5 (1 = poor, 2 = fair, 3 = good, 4 = very good, 5 = excellent) …………………….

**Acceptability/ Timeliness**

1. Are you willing to continue participating in surveillance system? Yes No
2. Any problem/hindrance to carrying out your work effectively? Yes No
3. Which of the hindrances, below affects sending data on regular basis?

***Tick all that applies***

a. Inadequate data capture tool. b. Complexity of the tools. c. Work overload. d. Lack of incentive. e. Inadequate knowledge on data use. f. others (specify)

1. How long does it take to collate health facility data from individual patient (in minutes)? a.1-10 b. 11-20 c. 20-30 d. >30minutes
2. How soon do you complete your monthly report within the new month? 1^st^ 5days end of 1^st^ week 2^nd^ week 3^rd^ week
3. Do you feel the system appreciates you for doing your job? Yes No
4. What will you need to do your job effectively? a. Incentives b. More staff training on data management c. Job aides d. others (specify)............................................
5. Have you ever made suggestions/comments about improving data reporting? Yes No
6. Was your suggestion taken? Yes No

**Sensitivity**

1. Are you satisfied with the outcomes of the malaria rapid kit diagnostic test? Yes No
2. Are there missing cases of malaria from the rapid kit test? Yes No
3. Are there misdiagnosed cases? Yes No
4. How will you rate the sensitivity (ability to correctly pick those with the disease) of the rapid kit test for malaria diagnosis? (a=95%, b=90%, c=80% d=70% e=60%)
5. Any suggestion for improvement? ………………………………………………………………………………………………

**Representativeness**

1. The data tools depict information on distribution of cases of malaria based on: a. Age b. Sex c. Location d. Outcome of disease e. Time of diagnosis ***(select as appropriate)***
2. Are private health facilities in your LGA sending reports? Yes No

**Planned use of data generated from the system/ data management:**

1. Do you collect data using IDSR 003 form? Yes No Not sure
2. What do you think is the usefulness of data collected at your facility (tick)? a. Analyzed and used for decision making b. Not analyzed but stored c. Not stored and not used d. don’t know
3. Does your data capture all age groups? Yes No
4. Do you get feedback from the State? Yes No Not sure
5. How often do you give feedback? Monthly Quarterly Yearly
6. Do you have stipends to carry out your work? Yes No
7. Stipends received are from: a. Government b. partners c. other source (please specify) ………………………………
8. Do you need more staff to assist with the data management? Yes No
9. Do you need more resources? Yes No
10. If inadequate, please tick resources needed: a. human b. financial c. materials for data capturing d. others (specify)...................
