# Supplementary material for: Evaluation of malaria surveillance system in Kano State, Nigeria, 2013–2016
Source: Infect Dis Poverty. 2020 Feb 10;9:15. doi: 10.1186/s40249-020-0629-2 (PMC7008566; doi:10.1186/s40249-020-0629-2)
Supplement: Supplementary file 2 — Additional file 2. Key Informant Interview Guide on Malaria Surveillance System Evaluation. [file 40249_2020_629_MOESM2_ESM.docx]

KEY INFORMANT INTERVIEW GUIDE

**EVALUATION OF MALARIA SURVEILLANCE SYSTEM IN KANO STATE**

1. Cadre of staff
2. Working experience in the Ministry (Years)

**Malaria Diagnosis and Treatment**

1. How often do you get the supply of malaria Rapid Diagnostic Kit (RDT)?
2. Are there guidelines for Management of uncomplicated /complicated malaria at the State? Yes / No

**Simplicity**

1. Considering your experience as a manager in malarial program in the State, kindly asses the simplicity of the data capture tools with respect to time required to complete filling each form and the volume of information required.
2. What were the feedback you got from the facility focal person and DSNOs concerning simplicity of the malaria screening test procedure?
3. Do you think the task shifting principle can be applied to malarial diagnostic test procedure? Yes No Not sure (If yes, what are your reasons)

**Flexibility**

1. As a major stakeholder, can you talk about any changes that have been effected in the malaria data capture tools and malaria treatment guidelines
2. What were the resources used to implement these changes? a. human b. financial c. none d. both
3. What has been the inputs of the results of previous supportive supervision on malaria surveillance system

**Data Quality**

1. Does the State have any form of data quality improvement training for the DSNOs and malaria focal persons? If yes, how often, and what modality
2. Is there regular supply of data management tools and RDT kits? How often are they supplied?
3. Does the State conduct supportive supervision to facilities? How? (is it as part of Integrated Supportive supervision or focus on malaria program)
4. Is there remarkable improvement in malaria data quality from the activity above?

**Sensitivity**

1. How would you rate the incidence of poorly treated malaria and severe cases of malaria? Are the RDT kits able to detect malaria cases?
2. How will you rate the sensitivity (ability to correctly pick those with the disease) of the rapid kit test for malaria diagnosis? (a=95%, b=90%, c=80% d=70% e=60%)

**Representativeness**

1. Are reports from all health facilities (Both private and public) captured in your monthly data? If not, why?
2. Are the data tools used in the state depict information on distribution of cases of malaria based on various variables such as age, sex, location, outcome of disease or time of diagnosis?

**Planned use of data generated from the system/ data management:**

1. How does the State manage the data generated from malaria surveillance?
2. How do you get the funding for malaria programs, and malaria diagnostic kits?
3. a. Government b. partners c. other sources (please specify) ……………………………… Please explain
4. Do you need more staff to assist with the data management? Yes / No

THANK YOU
